# Electrophiles against (Skin) Diseases: More Than Nrf2

**DOI:** 10.3390/biom10020271

**Published:** 2020-02-11

**Authors:** Paulina Hennig, Gabriele Fenini, Michela Di Filippo, Hans-Dietmar Beer

**Affiliations:** 1 Department of Dermatology, University Hospital of Zurich, Gloriastrasse 31, CH-8091 Zurich, Switzerland; Paulina.Hennig@usz.ch (P.H.); Gabriele.Fenini@usz.ch (G.F.); Michela.DiFilippo@usz.ch (M.D.F.); 2 Faculty of Medicine, University of Zurich, 8006 Zurich, Switzerland

**Keywords:** NRF2, skin, inflammation, inflammasomes, electrophile, DMF, NF-κB

## Abstract

The skin represents an indispensable barrier between the organism and the environment and is the first line of defense against exogenous insults. The transcription factor NRF2 is a central regulator of cytoprotection and stress resistance. NRF2 is activated in response to oxidative stress by reactive oxygen species (ROS) and electrophiles. These electrophiles oxidize specific cysteine residues of the NRF2 inhibitor KEAP1, leading to KEAP1 inactivation and, subsequently, NRF2 activation. As oxidative stress is associated with inflammation, the NRF2 pathway plays important roles in the pathogenesis of common inflammatory diseases and cancer in many tissues and organs, including the skin. The electrophile and NRF2 activator dimethyl fumarate (DMF) is an established and efficient drug for patients suffering from the common inflammatory skin disease psoriasis and the neuro-inflammatory disease multiple sclerosis (MS). In this review, we discuss possible molecular mechanisms underlying the therapeutic activity of DMF and other NRF2 activators. Recent evidence suggests that electrophiles not only activate NRF2, but also target other inflammation-associated pathways including the transcription factor NF-κB and the multi-protein complexes termed inflammasomes. Inflammasomes are central regulators of inflammation and are involved in many inflammatory conditions. Most importantly, the NRF2 and inflammasome pathways are connected at different levels, mainly antagonistically.

## 1. Introduction

The skin is the largest organ of the human body and represents its outermost barrier, which is in permanent contact with the environment [1]. It is frequently exposed to pathogens, such as bacteria and viruses, and other insults, for example, UV radiation [2]. The skin consists of two compartments, which are separated by the basement membrane. The inner compartment is the dermis, a connective tissue with extracellular matrix produced by fibroblasts and containing a collection of different immune cells. The outer structure is the epidermis, a stratified keratinized epithelium which is composed of several layers of keratinocytes in different stages of differentiation. In contrast to the dermis, the epidermis is a constantly renewing tissue. Under homeostatic conditions, the proliferation of keratinocytes is restricted to the basal cell layer and to hair follicles. Then, keratinocytes migrate outwards, terminally differentiate, lose their nucleus and form the stratum corneum, a structure particularly important for the protection of the skin. Epidermal keratinocytes not only form the outer structural barrier of the skin, but also express, in a constitutive or inducible manner, molecules, peptides and proteins which are actively involved in different defense and protective mechanisms [2].

The detection of stress factors by keratinocytes and other skin resident cells can result in the activation of different pathways. The transcription factor NRF2 (nuclear factor E2-related factor 2) is activated by oxidative and electrophilic stress and is involved in different processes in the skin, such as UV protection and would healing [3,4,5]. NRF2 induces the expression of cytoprotective proteins and enzymes, which support survival of cells, and represses the expression of pro-inflammatory cytokines [6,7]. Inflammation is associated with high levels of reactive oxygen species (ROS) and NRF2 is required for its termination. Strong stressors induce an inflammatory response and inflammation can be considered as an attempt of the tissue to restore homeostasis after its perturbation [8]. Inflammation is induced, among other things, upon the activation of inflammasomes, which are multi-protein complexes expressed by immune cells, such as macrophages and dendritic cells, but also by keratinocytes [9,10]. Inflammasomes detect many different stress factors and induce inflammation upon the regulation of activation and secretion of the proinflammatory cytokines (pro)interleukin(IL)-1β and -18.

The transcription factor NF-κB (nuclear factor κ light chain enhancer of activated B cells) plays a central role in homeostasis and inflammation in the skin [2,11,12] and participates in inflammasome and NRF2 regulation in a complex manner [13,14]. For example, although NF-κB is a positive regulator of proIL-1β expression, it restricts inflammasome activation and IL-1β secretion [15,16]. Recently, it was demonstrated that NRF2 possesses anti-inflammatory activity by the repression of proIL-1α and -β expression [6]. Moreover, the metabolite itaconate, which is increased in LPS(lipopolysaccharide)-activated macrophages, is an NRF2-activating electrophile and exerts anti-inflammatory activity by NRF2-dependent and NRF2-independent mechanisms [17,18].

Dimethyl fumarate (DMF) is a simple molecule and an approved drug for treatment of two different immune-mediated inflammatory diseases: psoriasis, which affects mainly the skin of 2%–4% of the population, and relapsing-remitting multiple sclerosis (RRMS), a common neurological disease [19,20]. Interestingly, DMF is an electrophile which activates the NRF2 pathway [21] and inhibits inflammasome activation [22] as well as NF-κB [23]. In this review, we discuss the different molecular mechanisms, which might underlie the pharmacological activity of DMF and other electrophiles in psoriasis, RRMS and other inflammatory (skin) diseases.

## 2. Redox Sensing and Redox Regulation

Reactive oxygen species (ROS) comprise a group of short-lived molecules generated in all cells by several pathways [24]. Due to electron transport chain leakage, mitochondria are a main source of ROS, as well as NADPH oxidases. Low levels of ROS are required for signaling, immune and other responses. However, high levels of ROS can result in the oxidation of several cellular molecules, including proteins, and are associated with inflammation and various human disorders, such as cardiovascular and neurodegenerative inflammatory diseases, and cancers [24,25].

To cope with oxidative stress, cells express ROS-detoxifying enzymes (see below). In addition, they are equipped with three different redox buffers which can scavenge ROS: the thioredoxin, the cysteine/cystine and the glutathione system, which are all based on the redox sensitivity of the amino acid cysteine [26,27]. Beyond classic thiol-disulfide bridges, the thiol group of cysteine can undergo a broad range of reversible and irreversible oxidations [27].

Glutathione is a cysteine-containing tripeptide and the predominant low molecular weight thiol found at a concentration of 1–10 mM in the cytoplasm [26]. The cytoplasmic ratio of reduced glutathione (GSH) to its oxidized form (GSSG) is up to 100:1, but in situations with oxidative stress and ROS, this ratio can drop to 1:1. Under these conditions, GSSG can oxidize accessible cysteine residues in cytoplasmic proteins. This reversible glutathionylation represents a type of post-translational modification and is believed to prevent other forms of irreversible oxidation, which might impair protein function, resulting in the disruption of cellular integrity and finally in cell death [28]. When the oxidative stress is over, GSH is removed from glutathionylated proteins by enzymatic and non-enzymatic mechanisms. Therefore, by protecting cysteine residues in proteins from irreversible oxidation and loss of function, glutathionylation represents a post-translational mechanism of defense against oxidative stress.

**Figure 1:**
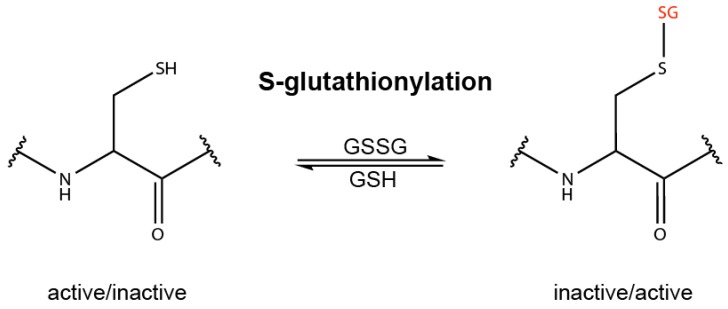
Redox regulation by oxidation of redox sensitive cysteine residues. The thiol group (SH) of specific cysteine residues of proteins can be oxidized by oxidized glutathione (GSSG) or other molecules, including cysteine-reactive electrophiles, causing S-glutathionylation or other types of cysteine oxidation, such as intermolecular disulfide bond formation. These types of post-translational modifications can influence the properties of proteins and the activity of enzymes, which is termed redox regulation or redox signaling.

Similar to irreversible oxidation, reversible glutathionylation might change the properties of proteins and enzymes as well (Figure 1). Although glutathionylation occurs at least mainly by non-enzymatic mechanisms, it is believed that it represents a well-controlled and site-specific event [27]. Therefore, glutathionylation can also be a type of redox regulation of proteins mediated by reversible post-translational modification of specific redox-sensitive regulatory cysteine residues. Prerequisites for glutathionylation and other types of oxidation are the accessibility of the thiol group of a cysteine on the protein surface and its low pKa, which enhances reactivity upon its deprotonation. Compared to the proteome, the number of glutathionylated proteins is supposed to be small and described as the «glutathionome» [29].

Redox regulation by glutathionylation has been demonstrated for several proteins, such as the NRF2 regulator KEAP1 (Kelch-like ECH-associated protein 1) (see below) [30,31], HIF-1α [32], p53 [33], actin, GAPDH [34], mitochondrial thymidine kinase 2 [35], caspase-3 [36,37], caspase-8 [38], caspase-1 [39], NLRP3 [40] and proIL-1β [41]. Moreover, redox regulation by other types of oxidation of redox-sensitive cysteine residues has been described, for example for p62 [42] or caspase-1 [43]. However, as the analytical detection of modified cysteine residues, particularly by glutathione but also by other molecules, is currently a major challenge in the field, the targeted cysteine residues are often unknown [34,35,38,40] or a matter of debate [36,37].

## 3. NRF2 is Activated by Electrophiles upon Cysteine Oxidation of its Inhibitor KEAP1

The NRF2 transcription factor is a central regulator of cytoprotection. In response to oxidative stress, NRF2 is activated by redox signaling upon oxidation of specific cysteine residues of its inhibitor KEAP1 [3,7,44]. At the transcriptional level, NRF2 expression is induced by NF-κB, a positive NRF2 feedback loop, the aryl hydrocarbon receptor and by several oncogenic pathways [45]. The transcriptional activity of NRF2 requires binding of sMAFs (small masculoaponeurotic fibrosarcoma) proteins to the carboxy terminal Neh1 (Nrf2-EHC homology) domain [46]. Then, NRF2 binds to AREs (antioxidant response elements) in the promoter region of about 250 target genes and regulates their transcription. Among them are many cytoprotective genes [19], whose products are essential for glutathione synthesis (e.g., glutamate-cysteine ligase), the thioredoxin system (e.g., thioredoxin reductase), detoxification (e.g., NAD(P)H dehydrogenase [quinone] 1), and drug excretion (e.g., multidrug resistance protein) [3]. Moreover, it was demonstrated that NRF2 can also repress gene expression by a yet incompletely characterized mechanisms [6]. Interestingly, these genes code for pro-inflammatory cytokines, such as proIL-1β, -α and IL-6. This finding represents an explanation of how NRF2 contributes directly to the resolution of inflammation. In addition, NRF2 activators inhibit also the transcriptional activity of NF-κB and the inflammasome pathway (see below) [19,22]. The expression and activity of NRF2 is very high in immune cells, which are frequently exposed to oxidative stress, particularly upon induction of the oxidative burst during phagocytosis of pathogens [47]. However, epithelial keratinocytes also express high levels of NRF2 and the transcription factor is critically involved in wound healing [4], UV protection [3,5], skin cancer development [48] and several severe autoimmune diseases with skin involvement, such as systemic lupus erythematosus, Sjogren’s syndrome and vitiligo [19]. Moreover, there is a lot of evidence—mainly based on experiments with NRF2 knockout mice—for roles of NRF2 in protection against many chronic inflammatory diseases, such as autoimmune diseases (e.g., MS), metabolic, respiratory, gastrointestinal, cardiovascular and particularly neurodegenerative diseases [3,19]. Although NRF2 activation is believed to prevent cancer development, the NRF2 pathway is frequently activated in different types of cancer and confers resistance to chemotherapy [3].

**Figure 2:**
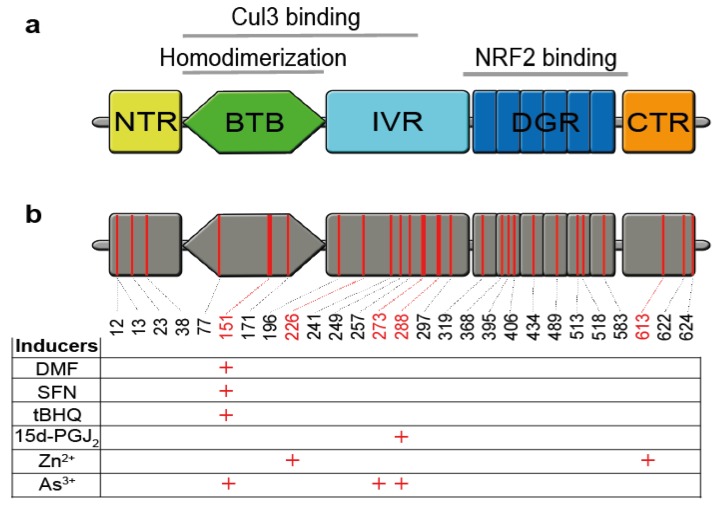
Structure of KEAP1 and the cysteine code. (**a**) Human KEAP1 is a 624 amino acid protein with five different domains. The BTB domain is required for KEAP1 dimerization and binding of Cul3. The DGR domain binds to the ETGE and DLG domain of NRF2. (**b**) The cysteine code of KEAP1. KEAP1 is a cysteine-rich protein and different electrophiles inhibit KEAP1 function by modification and oxidation of specific cysteine residues.

Under homeostatic conditions, the NRF2 protein possesses a half-life of only 10–30 min [45] and the KEAP1 adaptor protein is the main regulator of its stability and activity (Figure 2). Upon interaction of the BTB (Bric-à-Brac) domain, KEAP1 forms dimers which bind, via the Kelch domain, one molecule of NRF2 (one KEAP1 molecule interacts with the ETGE domain of NRF2, the second with the DLG domain) [44]. Moreover, KEAP1 binds the E3 ubiquitin ligase complex Cul3/Rbx1 (Cullin 3/RING-box protein 1) causing NRF2 ubiquitination and degradation via the proteasome pathway. Under homeostatic conditions, NRF2 is constantly degraded. However, small amounts of NRF2 might escape KEAP1-mediated degradation, allowing the translocation of NRF2 to the nucleus and, subsequently, a constitutive but weak expression of NRF2 target genes.

**Figure 3:**
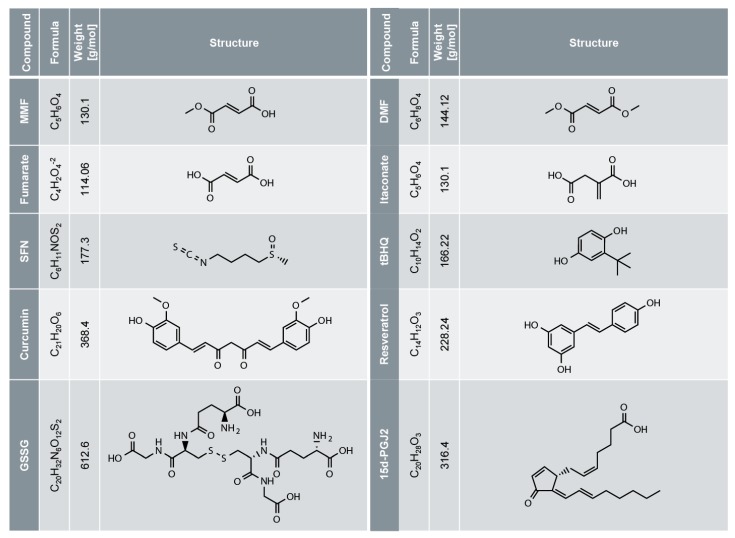
Structure of different NRF2-activating electrophiles. Name, formula, molar weight and structure of different electrophiles activating NRF2.

KEAP1 is a 624 amino acid protein with five domains and 27 cysteine residues (Figure 2) [44]. Some of these cysteine residues are redox sensitive and their oxidation by glutathione (Figure 1) or electrophiles (Figure 3) causes inactivation of KEAP1 (redox regulation). Then, newly synthesized NRF2 can directly enter the nucleus and induce NRF2 target gene expression, termed canonical NRF2 activation [3]. Interestingly, NRF2-activating electrophiles have different specificities for the cysteine residues of KEAP1, termed the cysteine code, although they all inactivate KEAP1 and therefore activate NRF2 (Figure 2) [3,44]. Several NRF2-activating compounds are endogenous molecules, such as 15d-PGJ2 (15-deoxy-Δ12,14-prostaglandin J2), itaconate or fumarate (Figure 3). 15d-PGJ2 is a naturally occurring cyclopentenone prostaglandin, which targets several pathways such as PPAR-γ (peroxisome proliferator-activated receptor-γ), and has anti-tumor, anti-inflammatory, anti-oxidation, anti-fibrosis, and anti-angiogenesis effects [49]. Itaconate is produced by macrophages in response to LPS treatment and has anti-inflammatory activity by NRF2-dependent and -independent effects [17,18]. Fumarate is a metabolite of the Krebs (or citric acid or tricarboxylic acid) cycle, and its accumulation, as reported in certain cancer cells, due to loss of function of fumarate hydratase, causes NRF2 activation [3]. Exogenous NRF2 activators are dimethyl fumarate (DMF), a cell-permeable derivative of fumarate, or Zn^2+^, which are used as an oral anti-inflammatory drug or in creams, respectively. Several NRF2 activators are found in plants used in traditional Chinese medicine and known for their anti-inflammatory activity [14], such as curcumin (a natural polyphenol found in the rhizome of *Curcuma* ssp.) [50]. The electrophile SFN (sulforaphane), a well-known and strong NRF2 activator, occurs in broccoli sprouts and resveratrol in red wine and grapes.

## 4. DMF for the Treatment of Psoriasis and Multiple Sclerosis (MS)

Dimethyl fumarate (DMF) is the main component of Fumaderm^®^ (Biogen Idec), a drug which was approved in 1994 in Germany for the treatment of patients suffering from severe psoriasis, and then in 2008 for moderate psoriasis. In 2017, a similar DMF product termed Skilarence^®^ (Almirall) was approved by the European Medicines Agency for moderate and severe psoriasis [20,51,52]. Psoriasis is a chronic T cell- and DC(dendritic cell)-mediated inflammatory skin disease affecting 2–4% of the population and plaque psoriasis is the most common type, affecting about 90% of all patients [53]. As about 30% of all patients suffer from moderate and severe psoriasis, there is a strong need for efficient systemic treatment options with few side effects. In 2012, two studies proved the efficacy of an oral delayed release formulation of DMF (BG-12, Tecfidera^®^) for the treatment of patients suffering from relapsing-remitting multiple sclerosis (RRMS), the most common type of MS [54,55]. MS is a chronic inflammatory autoimmune disease of the central nervous system triggered by autoreactive T cells against myelin, causing demyelination of axons and dendrites. Tissues affected by psoriasis and MS are infiltrated by Th1 and Th17 lymphocytes. DMF treatment causes a shift to a Th2 anti-inflammatory type response and DMF-treated patients have fewer circulating immune cells [51,52,56,57,58]. As a Th1/Th17 cytokine profile is also a characteristic of several other diseases, DMF may be also efficient in patients suffering from diseases such as colitis, Crohn’s disease, rheumatoid arthritis or systemic sclerosis [51,57,59]. Indeed, several clinical trials suggest the efficacy of DMF in patients suffering from other inflammatory diseases and cancer (Table 1). Patients tolerate DMF quite well, even in long-term treatment. Major adverse events are gastrointestinal (diarrhea, abdominal pain), skin flushing and, particularly, leukopenia, lymphopenia and eosinophilia, which have to be monitored regularly [20].

**Figure 4:**
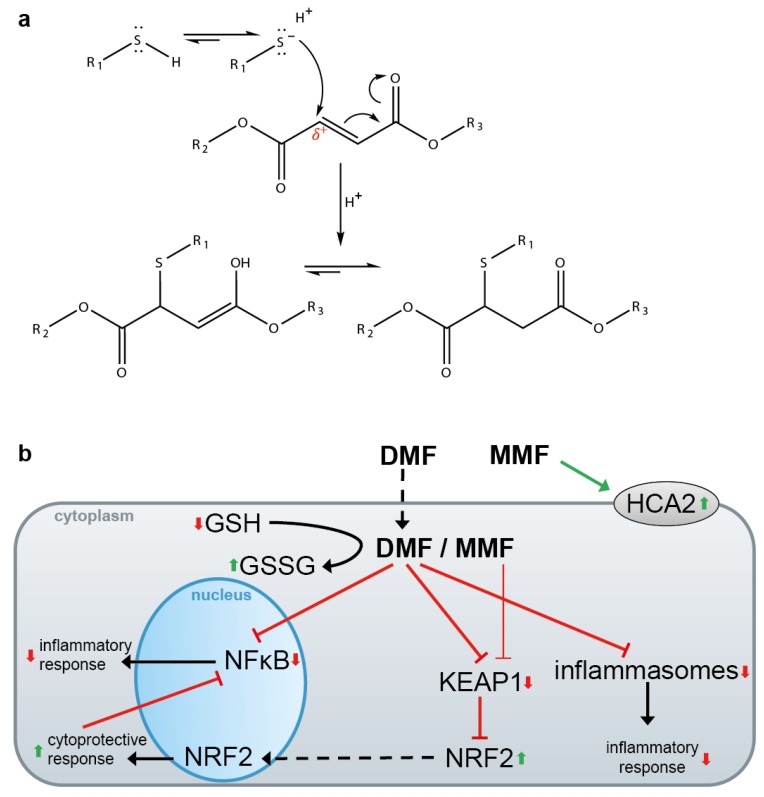
Proposed pathways targeted by DMF/MMF. (**a**) Michael addition of DMF/MMF and reactive cysteine residues in proteins. R_1_: protein or peptide; R_2_ and R_3_ = CH_3_: DMF; R_2_ = H and R_3_ = CH_3_: MMF; R_2_ and R_3_ = H: fumarate. (**b**) DMF and MMF target different pathways. MMF binds and activates the cell surface receptor HCA2. DMF and, to a lesser extent, MMF, enter the cell and activate NRF2 by the inhibition of KEAP1. DMF inhibits the NF-κB pathway by oxidation of specific cysteine residues in different NF-κB regulatory proteins. DMF can also inhibit inflammasome activation. Furthermore, DMF/MMF can react with GSH influencing the GSH/GSSG ratio, which in turn can affect the NRF2, NF-κB and inflammasome pathways.

DMF is a hydrophobic electrophile that easily enters cells across the plasma membrane and reacts with the sulfhydryl group of cysteine residues via the Michael addition (Figure 4a) in an irreversible manner [51]. DMF treatment has multiple effects in vitro (Figure 4b) [51]. However, the molecular mechanisms underlying the therapeutic activity of DMF in psoriasis and MS patients are poorly understood. DMF inactivates KEAP1 via oxidation of cysteine 151 [3] (Figure 2), causing nuclear translocation of NRF2, NRF2 target gene expression and repression of proinflammatory cytokine (see 3). Moreover, DMF inhibits NF-κB (see 5) and inflammasome activation (see 6). All these pathways antagonize inflammation, favoring a shift from a proinflammatory Th1/Th17 to a regulatory Th2 phenotype [57,58]. In addition, after entering the cells, DMF is trapped after reaction with GSH, free cysteine and thioredoxin (see 2) [51,60]. This causes a shift in the cellular redox potential, subsequent activation of NRF2 and inhibition of NF-κB and inflammasomes [39,58]. Other molecules and pathways that might be influenced by DMF in a direct or indirect manner include cAMP, HIF-1α, STATs, HO-1 and GAPDH [51,58].

However, DMF has only a half-life of 12 min in vitro and in vivo due to spontaneous and esterase-mediated hydrolysis, giving rise to monomethyl fumarate (MMF) (Figure 3) which possesses a half-life of 36 h [60,61]. Therefore, DMF is considered as a prodrug which is available after oral uptake only for a short time in the intestine, and in small amounts in the circulatory system, where it might influence immune cells [58]. Most likely, MMF is a (or the main, or even the only) therapeutically active substance in DMF-treated patients [60,61]. MMF, but not DMF, binds and activates the G_i_/G_0_ protein-coupled receptor HCA2 [60,62]. Interestingly, expression of HCA2 is required for a beneficial effect of DMF in experimental autoimmune encephalomyelitis (EAE), a mouse model of MS, although the underlying molecular mechanisms are not completely characterized [60,63]. Moreover, it has been concluded that HCA2 is the main mediator of MMF action, as MMF is negatively charged and not able—in contrast to the hydrophobic DMF—to translocate through the plasma membrane for targeting intracellular pathways such as the NRF2/KEAP1 pathway [60]. However, MMF is only a weak acid and, therefore, mainly an uncharged molecule. Most importantly, the activation of NRF2 by MMF in vitro and in vivo has been clearly demonstrated, although with less potency than DMF [58,64]. In addition, it has also been shown that NRF2 expression is required for a therapeutic effect of DMF in EAE and other mouse models of neuro-inflammation [65,66,67]. In conclusion, it is likely that DMF acts as a prodrug in psoriasis and MS patients. Its metabolite MMF is either the only therapeutically active compound or acts in concert with DMF. MMF has several targets—intracellularly and on the plasma membrane—in vitro and possibly also in vivo. However, as activation of the HCA2 receptor has been demonstrated only for MMF (and not for other electrophiles), which might underlie or contribute to the therapeutic activity of DMF/MMF in psoriasis and MS patients, DMF/MMF might have a unique efficacy in these patients, compared to other electrophiles which do not activate HCA2.

## 5. DMF and Other Electrophiles Inhibit the NF-κB Pathway

The transcription factor NF-κB plays fundamental roles in inflammation, cell survival, inflammatory diseases and cancer [68]. The p65 and p50 transcription factors of the NF-κB pathway are retained in the cytoplasm by the inhibitory protein IκBα. Activation of the IKK (IκB kinase) complex, consisting of IKKα, IKKβ and NEMO (NF-κB essential modulator), by stimulation of the cell—for example, by proinflammatory cytokines or TLRs—causes phosphorylation and subsequent proteasomal degradation of IκBα, translocation of p65/p50 to the nucleus, and target gene expression upon DNA binding [68]. DMF inhibits the NF-κB pathway at different levels. In 2002, it was shown that DMF blocks nuclear entry of activated NF-κB [23]. This seems be to partially mediated by MSK1 (mitogen stress-activated kinase 1), which is inhibited by DMF, causing reduced translocation and phosphorylation of p65 [69]. Interestingly, DMF antagonizes DC maturation by NF-κB inhibition, mediated by suppression of ERK1/2 and MSK1 signaling [69]. DMF (but not MMF) can also reduce DNA binding of p65 caused by the decreased phosphorylation of p65 at Ser276 by MSK1 and MSK2 [70,71,72]. Moreover, it has been shown that DMF directly modifies p65 at Cys38 and thereby inhibits its translocation and DNA binding [73]. DMF rather than MMF targets and oxidizes the redox scavenger Trx1 (thioredoxin 1) at Cys73 [74]. As Trx1 is required for the reduction in cysteine residues in the DNA-binding domain of NF-κB, Trx1, oxidized by DMF, antagonizes NF-κB and shifts cancer cells towards death [74]. Similarly, high concentrations of DMF (but not MMF) cause cell death of CTCL (cutaneous T-cell lymphoma) cells by the inhibition of NF-κB, whereas bystander T cells are left unaffected [75]. Nevertheless, the drop in lymphocyte subpopulations in patients treated with DMF is most likely at least partially caused by direct and indirect NF-κB inhibition mediated by DMF and MMF [58,76]. It has been reported that NF-κB inhibition by DMF (but not by MMF) occurs by NRF2-independent mechanisms [77], but also by those requiring NRF2 expression and activity [58]. For example, HO-1, which is an NRF2 target gene [78], binds and inhibits NF-κB in the nucleus [58,79]. Moreover, the NRF2 and NF-κB pathways are coupled in a complex manner [13,14]. For example, the interaction of KEAP1 with IKKβ causes its degradation and NF-κB suppression [80,81]. In conclusion, DMF and, to a lesser extent, MMF, target and inhibit the NF-κB pathway by different NRF2-independent and -dependent mechanisms. Consequently, both pathways contribute to cytoprotection in vitro but also in vivo, as shown in a mouse model of Parkinson’s disease [82,83].

The NF-κB pathway is also a well characterized example of redox regulation by gluathionylation [84]. p65 is directly inhibited upon modification by glutathione [85], but glutathione can also oxidize IκBα at Cys189, causing its stabilization and indirect NF-κB inhibition [86]. Similarly, SFN blocks NF-κB activity by several mechanisms [87,88]. SFN oxidizes IκB [89], thereby inhibiting its phosphorylation and downstream NF-κB activation, but also targets specific cysteine residues of p50/p65, causing a reduction in DNA binding [90]. In addition, more indirect effects have also been suggested. SFN induces HO-1 expression via NRF2, which in turn inhibits NF-κB [91]. The isothiocyanate can also react with and oxidize components of cellular redox buffers, such as glutathione and thioredoxin, which are required to retain NF-κB’s DNA-binding capacity [87,92]. Moreover, there is evidence for an inhibition of NF-κB by several other NRF2-activating electrophiles, particularly by 15d-PGJ2 [78]. 15d-PGJ2 is a strong endogenous electrophile which targets the NF-κB pathway independently of its receptor PPARγ [93]. 15d-PGJ2 oxidizes a specific cysteine residue of IκB, causing its stabilization [94,95]. In addition, 15d-PGJ2 is able to react with Cys62 of p50 [96] and also with p65 [97] resulting in the inhibition of NF-κB activity.

In summary, NF-κB is inhibited by several NRF2-activating electrophiles, mainly independently of NRF2 activity, by directly targeting specific regulatory cysteine residues in different components of the NF-κB pathway.

## 6. DMF and Other Electrophiles Inhibit Inflammasome Activation

In 2011, Freigang et al. demonstrated an unexpected crosstalk between NRF2 and the inflammasome pathway in a mouse model of atherosclerosis [98]. Meanwhile, more than 100 publications confirmed the complex and poorly understood relationship of both stress-activated pathways (reviewed in [14]).

**Figure 5:**
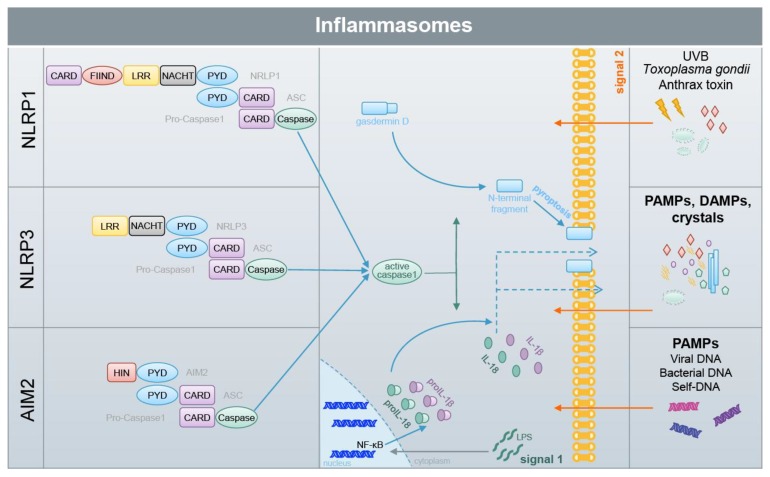
Inflammasomes are activated by different stress factors. NLRP1, NLRP3 and AIM2 represent important inflammasome sensor proteins, which consist of different domains. Upon detection of several different stress factors (PAMPs and DAMPs), inflammasomes are assembled, which is mediated by homotypic interactions of the pyrin (PYD) domain and the CARD. ASC is an adaptor protein which mediates the binding of pro-caspase-1 to the inflammasome sensor. Inflammasome assembly induces caspase-1 activation, which in turn cleaves and thereby activates gasdermin D and proIL-1β and -18. The N-terminal gasdermin D fragment forms pores in the outer membrane, allowing the release of IL-1β and -18 and inducing pyroptosis, a lytic type of cell death. IL-1β and -18 are strong proinflammatory cytokines, inducing inflammation in vivo. Particularly, activation of the NLRP3 and AIM2 inflammasome requires priming (signal 1) which induces the expression of inflammasome and inflammasome-associated proteins, such as NLRP3, AIM2 and proIL-1β.

Inflammasomes comprise a group of protein complexes which assemble upon the detection of different stress factors (Figure 5) [10,99]. They consist of a sensor protein, such as NLRP3 (nucleotide-binding oligomerization domain (NOD)-like receptor containing pyrin domain 3), NLRP1 or AIM2 (absent in melanoma 2), the adaptor protein ASC (apoptosis-associated speck-like protein containing a CARD [caspase activation and recruitment domain]) and the effector molecule caspase-1, a cysteine protease [100]. The complexes are held together by homotypic interactions of the death domain fold members the CARD and pyrin domain [101]. The sensor proteins detect exogenous pathogens or endogenous stress factors, termed pathogen-associated molecular patterns (PAMPs) and danger-associated molecular patters (DAMPs) [102]. This induces the formation of large ASC complexes, termed ASC specks, and, in turn, the activation of caspase-1. Then, caspase-1 cleaves and activates the proinflammatory cytokines proIL(interleukin)-1β and -18, which induce inflammation upon their release (Figure 5) [103]. Interestingly, IL-1β and -18 lack a signal peptide for secretion by the canonical ER(endoplasmic reticulum)-Golgi-dependent pathway and are released by one or several incompletely understood pathways, collectively termed unconventional protein secretion [104]. The secretion of IL-1β and -18 is supported by the cleavage of gasdermin D by caspase-1. The amino terminal fragment of gasdermin D forms pores in the outer membrane upon oligomerization, supporting the release of the proinflammatory cytokines [105,106]. In addition, pore formation causes water influx, a swelling of the cell, and finally its rupture. This lytic type of cell death is termed pyroptosis [107]. However, inflammasome activation does not necessarily cause pyroptosis, even when IL-1β and -18 are released [108,109,110,111,112]. Inflammasomes play fundamental roles in immunity but are also associated with many autoimmune, autoinflammatory, metabolic and infectious diseases [99,100]. Particularly, NLRP3 is believed to be critically involved in common diseases, whereas its role in immunity is rather minor [113,114]. Therefore, the mechanisms underlying NLRP3 inflammasome activation are of high medical interest. Two signals are required for NLRP3 activation. The first is mediated by LPS (or tumor necrosis factor (TNF)α, or other stimuli causing NF-κB activation), which binds and activates TLR4 (toll-like receptor) in immune cells, causing, in an NF-κB-dependent manner, the transcriptional upregulation of NLRP3, proIL-1β and -18 expression [115]. NLRP3 activating PAMPs and DAMPs represent “signal two”. However, how PAMPs and DAMPs induce NLRP3 inflammasome activation at the molecular level is completely understood. Among others, a role of ROS, which are associated with NLRP3-activating PAMPs and DAMPs, is being discussed [102,116]. NLRP3 might sense changes in ROS via TXNIP (thioredoxin-interacting protein) [117], which is bound to thioredoxin under homeostatic conditions, or via MAVS (mitochondrial antiviral signaling protein) [118]. In contrast, the NLRP3 inflammasome is inhibited in immune cells of SOD1 (superoxide dismutase 1)-deficient mice which have strongly increased levels of ROS [39]. Therefore, it should be pointed out that the role of ROS in NLRP3 activation is a matter of debate and could be species-, cell type-, context- or/and stimulus-dependent [119].

ROS play a role in atherosclerosis, where they cause oxidation of lipids and their accumulation in the arterial wall [98]. This is also true for IL-1β [120]. Surprisingly, mice lacking expression of NRF2 are protected from atherosclerosis [98,121]. Moreover, NRF2 knockout mice are also protected from disease in a model of chronic kidney disease [122]. Interestingly, both disease models depend on inflammasome activation, and the ablation of NRF2 expression dampens inflammasome activation [22,123,124]. The underlying mechanisms are unknown [14].

DMF is an effective drug for patients suffering from RRMS or psoriasis (see 4.) and several publications suggest the involvement of the inflammasome pathway in both inflammatory diseases. The NLRP3 inflammasome plays a role in EAE, a mouse model of MS [125,126,127] and pharmacological targeting of NLRP3 ameliorates EAE [128,129]. Moreover, there is also evidence for a function of NLRP3 in MS in humans [130]. In contrast, several inflammasome sensors are involved in psoriasis. Sequence variants of the *NLRP1* gene are associated with psoriasis [131,132]. This effect might be mediated by keratinocytes, as NLRP1 is the central inflammasome sensor in this cell type [133,134]. Moreover, the AIM2 inflammasome in keratinocytes also supports the development of psoriasis in humans [135], and even NLRP3 variants seem to play a role in psoriasis susceptibility [136]. In addition, components of the inflammasome pathway are upregulated in psoriatic epidermis [137]. In contrast, in a murine model of psoriasis the inflammasome pathway has only an effect upon ablation in immune cells, but not in keratinocytes [138], and the targeting of NLRP3 ameliorates imiquimod-induced psoriasis [139]. However, it is known that the inflammasome pathway in keratinocytes is not conserved between humans and mice [140]. There is a lot of evidence that not only DMF, but also other NRF2-activating electrophiles, are beneficial in many inflammatory disease models, which are associated with inflammasome activation (reviewed in [14]). This is (at least partially) mediated by the KEAP1/NRF2 pathway (see 3.) and by inhibition of NF-κB (see 5.). However, there is also evidence that electrophiles can directly inhibit inflammasome activation [14]. SFN and 15d-PGJ2 inhibit activation of NLRP3 in the absence of NRF2 expression in a very fast manner, suggesting that transcriptional effects are not relevant for NLRP3 inhibition [22,141,142]. SFN inhibits NLRP3 even in KEAP1 knockout cells [22]. DMF has beneficial effects in EAE in the absence of NRF2 [143] and blocks different types of inflammasomes in different cell types [22,144]. Moreover, SFN also inhibits the activation of different types of inflammasomes, and neither NRF2 target gene expression nor protein synthesis in general are required for this effect [22,141]. All these results demonstrate that electrophiles can inhibit the inflammasome pathway in a direct manner, perhaps via the modification of reactive cysteine residues of inflammasome proteins or those which regulate activation of these complexes. However, the molecular mechanisms underlying inflammasome inhibition by electrophiles are incompletely characterized [14,141].

## 7. Conclusions and Outlook

The electrophile DMF is used as a rather safe and well established drug for the treatment of patients suffering from the common inflammatory skin disease psoriasis [20] or from the neurological disorder RRMS [54,55]. Moreover, DMF is being tested for pharmacological activity in several other inflammatory (skin) conditions [51,57,59]. Other electrophiles, occurring in plants and known in traditional Chinese medicine for their anti-inflammatory activity, such as SFN, are also being extensively tested for therapeutic activity in different inflammatory conditions, particularly in neurological diseases, but also for the prevention and treatment of different types of cancer [19,151,152,153].

DMF, SFN and many other electrophiles react with accessible cysteine residues of proteins with a low pK_a_ value. These covalent modifications can change the properties of proteins, termed redox regulation. This is well known for reversible modifications of cysteine residues by oxidized glutathione under conditions characterized by oxidative stress, and ROS associated with inflammatory conditions [27,28,29]. The NRF2 inhibitor KEAP1 is a well known example of such a redox sensor [3], which is inactivated upon oxidation of specific cysteine residues by electrophiles, allowing the liberation of the cytoprotective NRF2 transcription factor and induction of NRF2 target gene expression (see 3.). It is generally accepted that NRF2 activation has anti-inflammatory activity and is beneficial for patients suffering from inflammatory conditions. However, NRF2-activating electrophiles do also react with cysteine residues of other proteins, changing their properties. A well-established example is the NF-κB pathway, which is inhibited by targeting different proteins by electrophiles (see 5.). More recently, a crosstalk between NRF2-activating and NF-κB-inhibiting electrophiles with inflammasome complexes has been established (see 6.). Although the molecular targets are not identified yet, the fact that inflammasome activation is associated with many inflammatory conditions, including psoriasis and MS, also suggests the contribution of inflammasome inhibition to the therapeutic activity of electrophiles. This is supported by several publications demonstrating a strong, fast and efficient inhibition of inflammasome activation by all tested NRF2 activators, particularly in vitro but also in vivo. In this context, it is tempting to speculate that the endogenous anti-inflammatory electrophile itaconate is not only a NRF2 activator, but inhibits inflammasomes and NF-κB directly [17,18]. Moreover, it should be kept in mind that, in addition to KEAP1 and components of the NF-κB and of the inflammasome pathway, several other proteins exist which are regulated by glutathione modification. It may well be that these proteins are also modified and regulated by other electrophiles (see 2.).

Although the existing literature suggests that electrophiles such as SNF, 15d-PGJ2 and DMF target the same pathways, it cannot be expected that they do it with the same efficacy and specificity, particularly in vivo [29]. For example, DMF is known to be unstable in vitro and in vivo. Its main (stable) metabolite MMF has more polar properties and, therefore, cannot enter cells as efficiently as DMF. However, cell penetration of electrophiles is required for targeting the NRF2, NF-κB and inflammasome pathway. DMF seems to be unique in the group of electrophiles, as only its metabolite, MMF, is known to activate the cell surface receptor HCA2 [62,63]. Vice versa, it might well be that the other electrophiles have, in addition to the common targets, also specific, but currently unknown, targets. Most importantly, due to steric and other structural and chemical reasons, it can be expected that each electrophile possesses a certain specificity for the many known (and unknown) cysteine targets in the different redox regulated proteins and pathways [27,29]. The characterization of the protein and pathway specificity of cysteine-targeting electrophiles will be a task for the future. This might not only allow the determination of the molecular targets of the existing pharmacologically used electrophiles, such as DMF and SFN, but also the development of new tailor-made molecules and drugs for the many inflammatory conditions which are associated with NRF2, NF-κB and inflammasomes.

## Figures and Tables

**Table 1:** Clinical trials with DMF for treatment of patients suffering from diseases other than psoriasis and RRMS.

Condition	CTI	St	n	Ph	Design	Outcome	Ref
**Obstructive Sleep Apnea**	NCT02438137	C	65	2	Rd-2B-P	Partial response	[145]
**Adult Brain Glioblastoma**	NCT02337426	C	12	1	O	SafePhase 2 under consideration	[146]
**Rheumatoid Arthritis**	NCT00810836	C	153	2	Rd-2B-P	Not effective	[147]
**Cutaneous Lupus Erythematosus**	NCT01352988	C	11	2	O	Safe and effectiveRandomize trial required	[148]
**Chronic Lymphocytic Leukemia**	NCT02784834	T	2	1	O	1/2 patients lack of efficacy	
**Systemic Sclerosis**	NCT02981082	R	34	1	Rd-4B-P		[149,150]
**Cutaneous T Cell Lymphoma**	NCT02546440	R	25	2	O		[75]

CTI, ClinicalTrial.gov Identifier; St, status of the trial; C, completed; T, terminated; R, recruiting; n, number of enrolled individuals; Ph, phase; Rd, randomized; O, open-label; 2B, double-blind; 4B, quadruple-blind; P, placebo-controlled.
